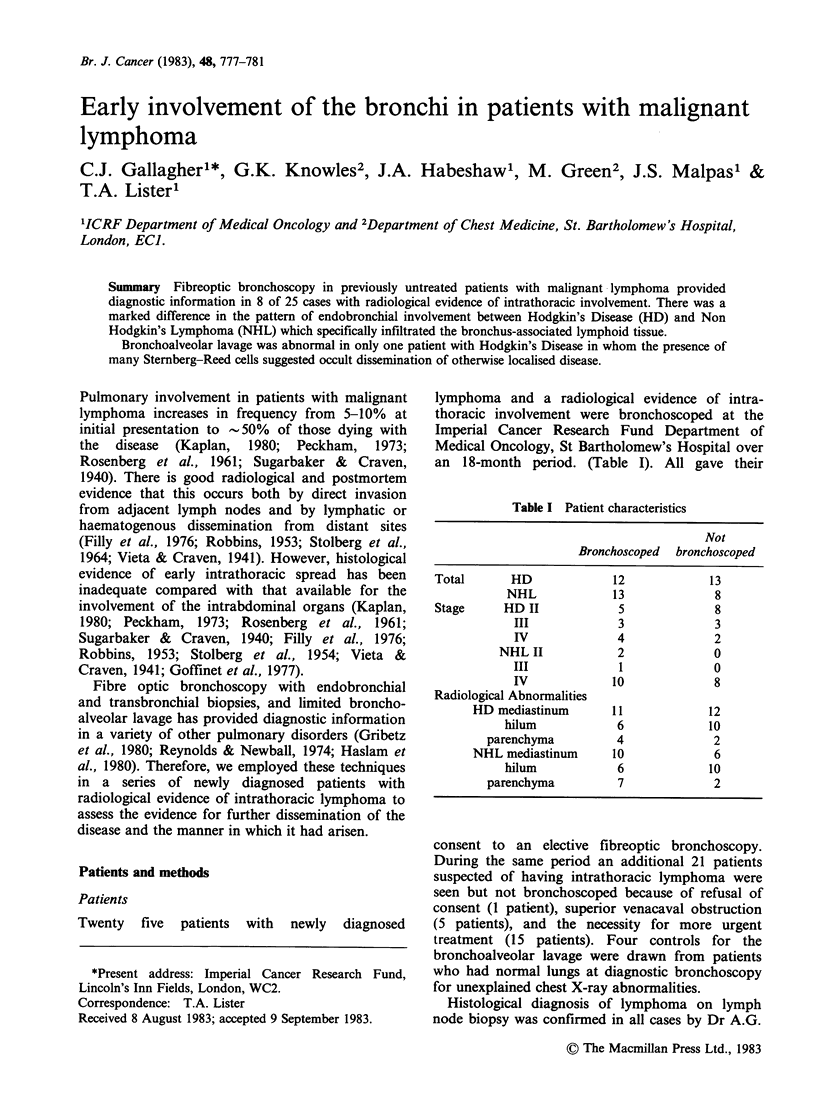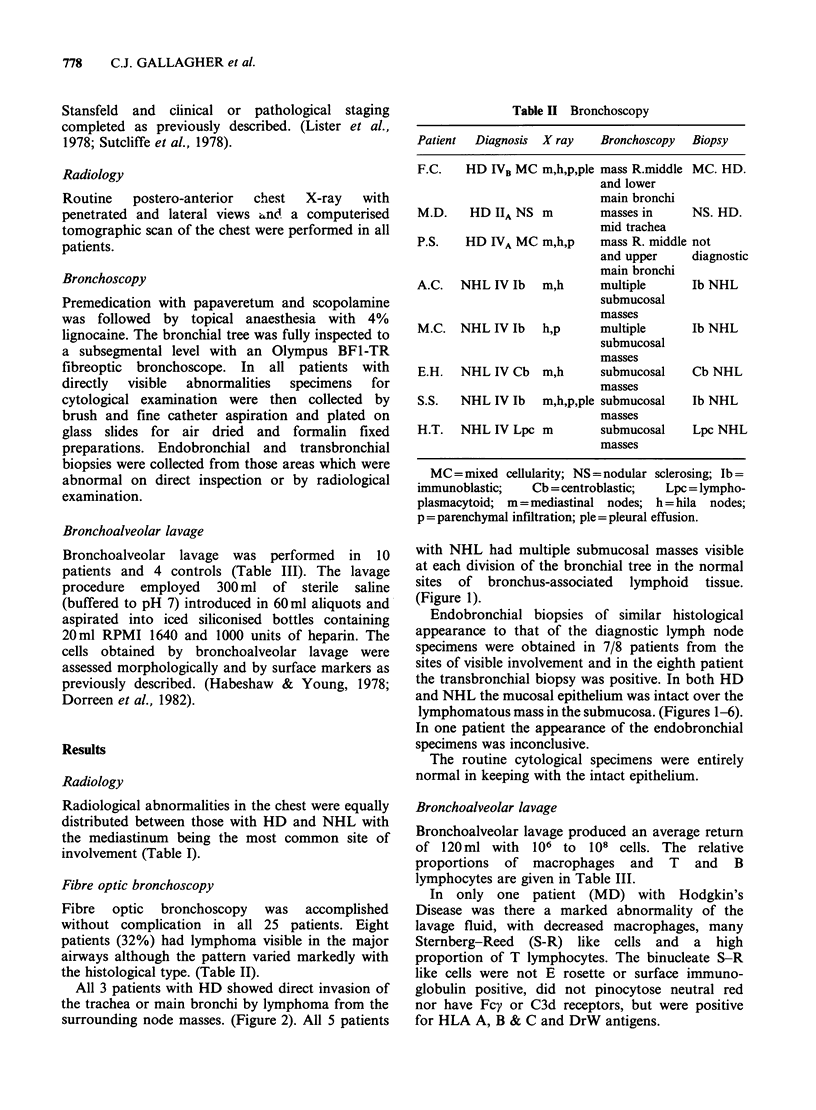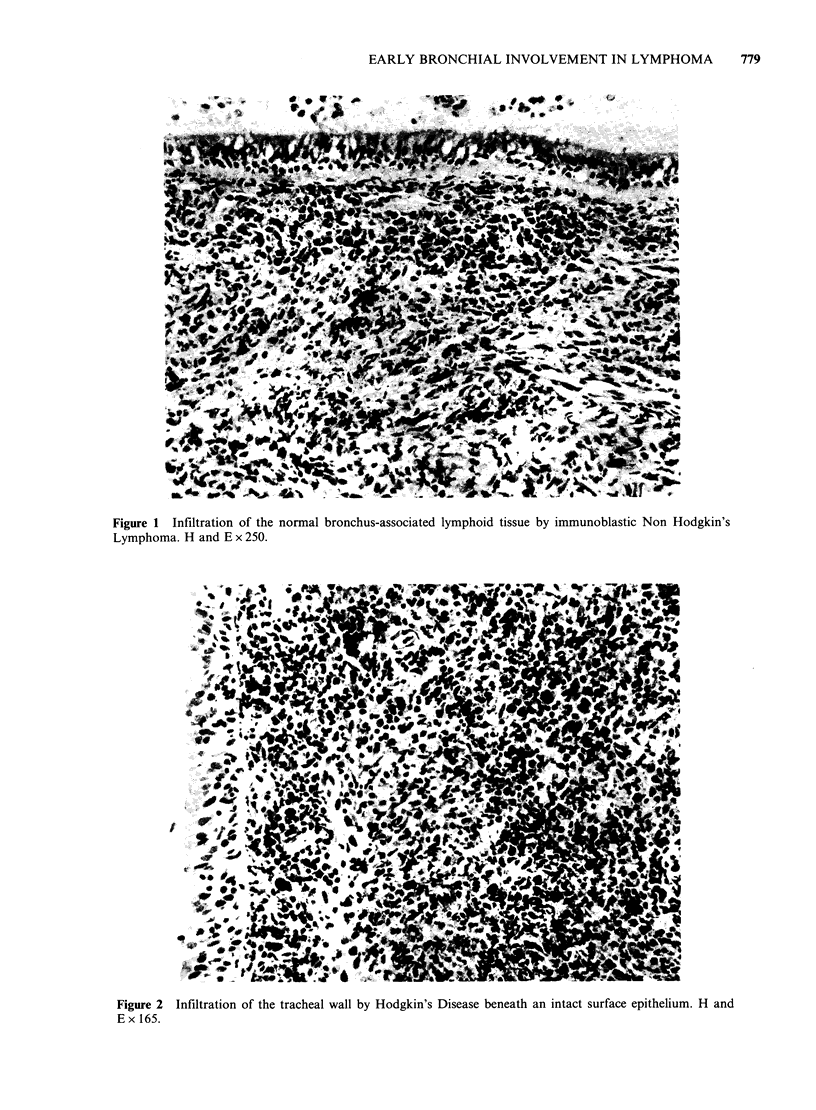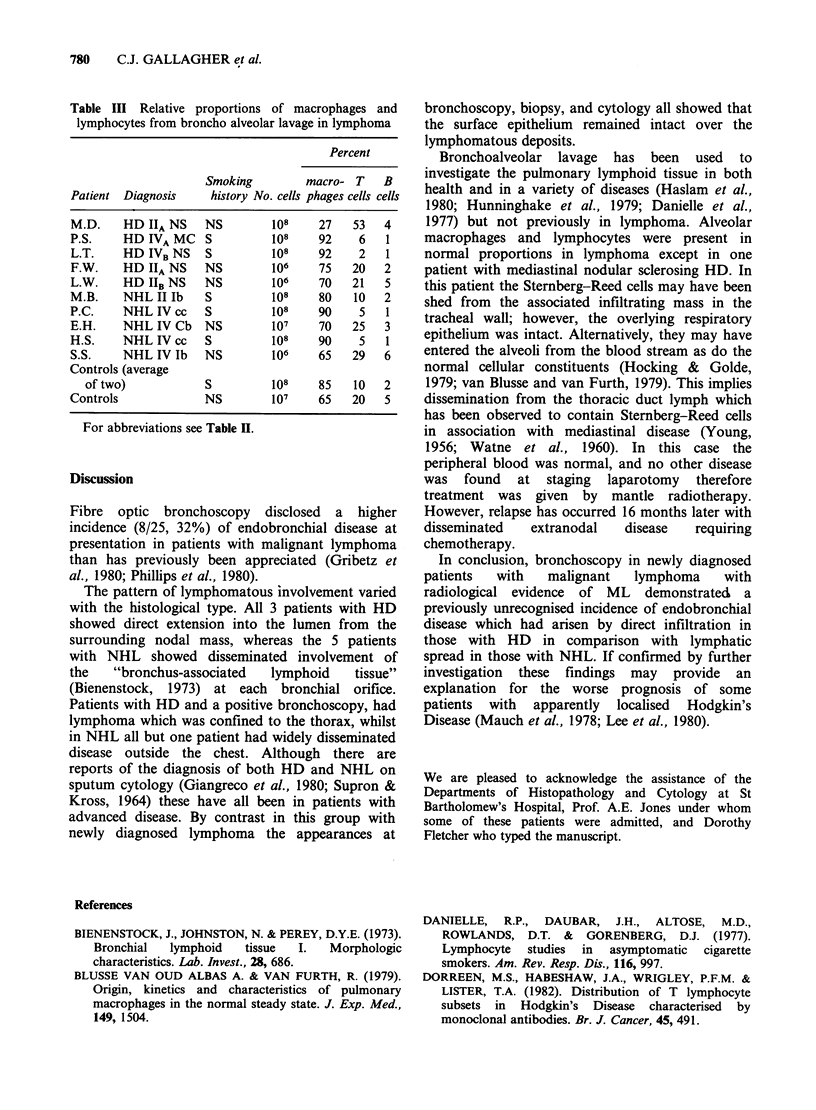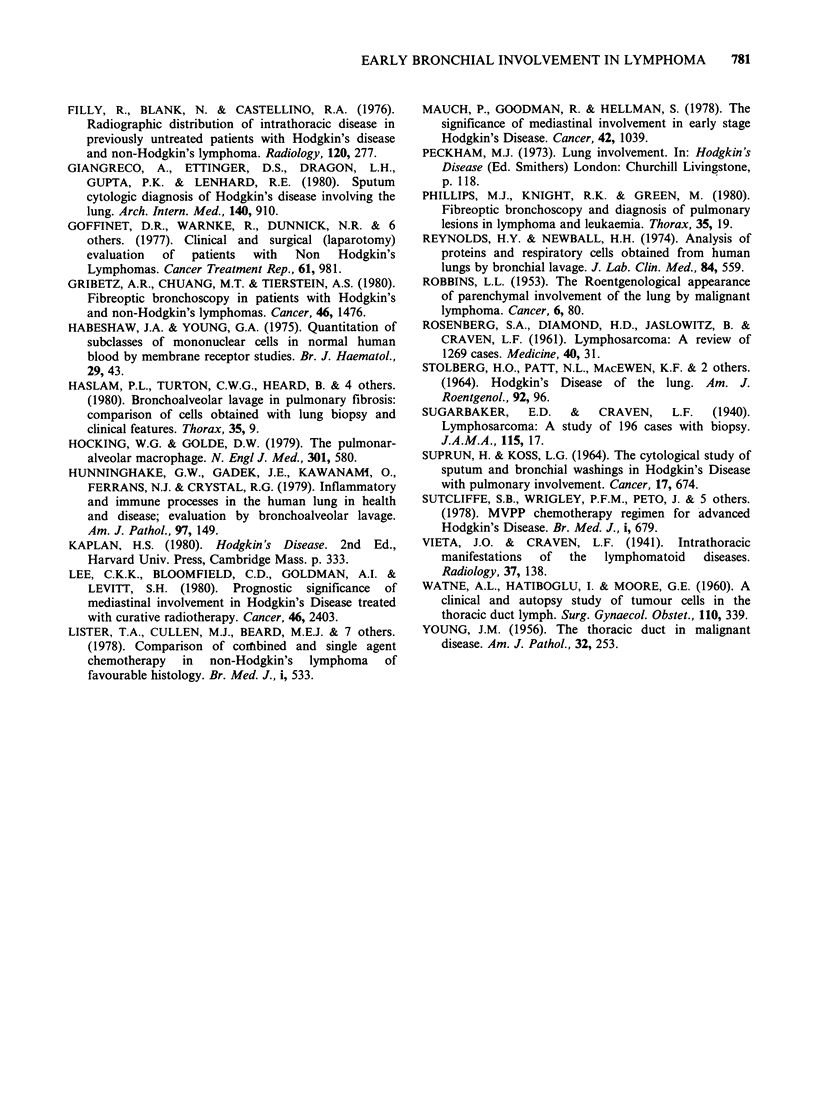# Early involvement of the bronchi in patients with malignant lymphoma.

**DOI:** 10.1038/bjc.1983.266

**Published:** 1983-12

**Authors:** C. J. Gallagher, G. K. Knowles, J. A. Habeshaw, M. Green, J. S. Malpas, T. A. Lister

## Abstract

**Images:**


*Br. J. Cancer* (1983), **48**, 777–781

### Early involvement of the bronchi in patients with malignant lymphoma

C.J. Gallagher[1]*, G.K. Knowles[2], J.A. Habeshaw[1], M. Green[2], J.S. Malpas[1] & T.A. Lister[1]

[1]*ICRF Department of Medical Oncology and* [2]*Department of Chest Medicine, St. Bartholomew's Hospital, London, EC1.*

**Summary** Fibreoptic bronchoscopy in previously untreated patients with malignant lymphoma provided diagnostic information in 8 of 25 cases with radiological evidence of intrathoracic involvement. There was a marked difference in the pattern of endobronchial involvement between Hodgkin's Disease (HD) and Non Hodgkin's Lymphoma (NHL) which specifically infiltrated the bronchus-associated lymphoid tissue.

Bronchoalveolar lavage was abnormal in only one patient with Hodgkin's Disease in whom the presence of many Sternberg–Reed cells suggested occult dissemination of otherwise localised disease.

Pulmonary involvement in patients with malignant lymphoma increases in frequency from 5–10% at initial presentation to ~50% of those dying with the disease (Kaplan, 1980; Peckham, 1973; Rosenberg *et al.*, 1961; Sugarbaker & Craven, 1940). There is good radiological and postmortem evidence that this occurs both by direct invasion from adjacent lymph nodes and by lymphatic or haematogenous dissemination from distant sites (Filly *et al.*, 1976; Robbins, 1953; Stolberg *et al.*, 1964; Vieta & Craven, 1941). However, histological evidence of early intrathoracic spread has been inadequate compared with that available for the involvement of the intrabdominal organs (Kaplan, 1980; Peckham, 1973; Rosenberg *et al.*, 1961; Sugarbaker & Craven, 1940; Filly *et al.*, 1976; Robbins, 1953; Stolberg *et al.*, 1954; Vieta & Craven, 1941; Goffinet *et al.*, 1977).

Fibre optic bronchoscopy with endobronchial and transbronchial biopsies, and limited broncho-alveolar lavage has provided diagnostic information in a variety of other pulmonary disorders (Gribetz *et al.*, 1980; Reynolds & Newball, 1974; Haslam *et al.*, 1980). Therefore, we employed these techniques in a series of newly diagnosed patients with radiological evidence of intrathoracic lymphoma to assess the evidence for further dissemination of the disease and the manner in which it had arisen.

### Patients and methods

### *Patients*

Twenty five patients with newly diagnosed lymphoma and a radiological evidence of intra-thoracic involvement were bronchoscoped at the Imperial Cancer Research Fund Department of Medical Oncology, St Bartholomew's Hospital over an 18-month period. (Table I). All gave their consent to an elective fibreoptic bronchoscopy. During the same period an additional 21 patients suspected of having intrathoracic lymphoma were seen but not bronchoscoped because of refusal of consent (1 patient), superior venacaval obstruction (5 patients), and the necessity for more urgent treatment (15 patients). Four controls for the bronchoalveolar lavage were drawn from patients who had normal lungs at diagnostic bronchoscopy for unexplained chest X-ray abnormalities.

Histological diagnosis of lymphoma on lymph node biopsy was confirmed in all cases by Dr A.G.

**Table I** Patient characteristics

| | | *Bronchoscoped* | *Not bronchoscoped* |
|---|---|---|---|
| Total | HD | 12 | 13 |
| | NHL | 13 | 8 |
| Stage | HD II | 5 | 8 |
| | III | 3 | 3 |
| | IV | 4 | 2 |
| | NHL II | 2 | 0 |
| | III | 1 | 0 |
| | IV | 10 | 8 |
| Radiological Abnormalities | | | |
| | HD mediastinum | 11 | 12 |
| | hilum | 6 | 10 |
| | parenchyma | 4 | 2 |
| | NHL mediastinum | 10 | 6 |
| | hilum | 6 | 10 |
| | parenchyma | 7 | 2 |

*Present address: Imperial Cancer Research Fund, Lincoln's Inn Fields, London, WC2.
Correspondence: T.A. Lister
Received 8 August 1983; accepted 9 September 1983.

© The Macmillan Press Ltd., 1983

Stansfeld and clinical or pathological staging completed as previously described. (Lister *et al.*, 1978; Sutcliffe *et al.*, 1978).

*Radiology*

Routine postero-anterior chest X-ray with penetrated and lateral views and a computerised tomographic scan of the chest were performed in all patients.

*Bronchoscopy*

Premedication with papaveretum and scopolamine was followed by topical anaesthesia with 4% lignocaine. The bronchial tree was fully inspected to a subsegmental level with an Olympus BF1-TR fibreoptic bronchoscope. In all patients with directly visible abnormalities specimens for cytological examination were then collected by brush and fine catheter aspiration and plated on glass slides for air dried and formalin fixed preparations. Endobronchial and transbronchial biopsies were collected from those areas which were abnormal on direct inspection or by radiological examination.

*Bronchoalveolar lavage*

Bronchoalveolar lavage was performed in 10 patients and 4 controls (Table III). The lavage procedure employed 300 ml of sterile saline (buffered to pH 7) introduced in 60 ml aliquots and aspirated into iced siliconised bottles containing 20 ml RPMI 1640 and 1000 units of heparin. The cells obtained by bronchoalveolar lavage were assessed morphologically and by surface markers as previously described. (Habeshaw & Young, 1978; Dorreen *et al.*, 1982).

### Results

*Radiology*

Radiological abnormalities in the chest were equally distributed between those with HD and NHL with the mediastinum being the most common site of involvement (Table I).

*Fibre optic bronchoscopy*

Fibre optic bronchoscopy was accomplished without complication in all 25 patients. Eight patients (32%) had lymphoma visible in the major airways although the pattern varied markedly with the histological type. (Table II).

All 3 patients with HD showed direct invasion of the trachea or main bronchi by lymphoma from the surrounding node masses. (Figure 2). All 5 patients with NHL had multiple submucosal masses visible at each division of the bronchial tree in the normal sites of bronchus-associated lymphoid tissue. (Figure 1).

**Table II** Bronchoscopy

| *Patient* | *Diagnosis* | *X ray* | *Bronchoscopy* | *Biopsy* |
|---|---|---|---|---|
| F.C. | HD $IV_B$ MC | m,h,p,ple | mass R.middle and lower main bronchi | MC. HD. |
| M.D. | HD $II_A$ NS | m | masses in mid trachea | NS. HD. |
| P.S. | HD $IV_A$ MC | m,h,p | mass R. middle and upper main bronchi | not diagnostic |
| A.C. | NHL IV Ib | m,h | multiple submucosal masses | Ib NHL |
| M.C. | NHL IV Ib | h,p | multiple submucosal masses | Ib NHL |
| E.H. | NHL IV Cb | m,h | submucosal masses | Cb NHL |
| S.S. | NHL IV Ib | m,h,p,ple | submucosal masses | Ib NHL |
| H.T. | NHL IV Lpc | m | submucosal masses | Lpc NHL |

MC = mixed cellularity; NS = nodular sclerosing; Ib = immunoblastic; Cb = centroblastic; Lpc = lymphoplasmacytoid; m = mediastinal nodes; h = hila nodes; p = parenchymal infiltration; ple = pleural effusion.

Endobronchial biopsies of similar histological appearance to that of the diagnostic lymph node specimens were obtained in 7/8 patients from the sites of visible involvement and in the eighth patient the transbronchial biopsy was positive. In both HD and NHL the mucosal epithelium was intact over the lymphomatous mass in the submucosa. (Figures 1–6). In one patient the appearance of the endobronchial specimens was inconclusive.

The routine cytological specimens were entirely normal in keeping with the intact epithelium.

*Bronchoalveolar lavage*

Bronchoalveolar lavage produced an average return of 120 ml with $10^6$ to $10^8$ cells. The relative proportions of macrophages and T and B lymphocytes are given in Table III.

In only one patient (MD) with Hodgkin's Disease was there a marked abnormality of the lavage fluid, with decreased macrophages, many Sternberg–Reed (S-R) like cells and a high proportion of T lymphocytes. The binucleate S–R like cells were not E rosette or surface immunoglobulin positive, did not pinocytose neutral red nor have Fcγ or C3d receptors, but were positive for HLA A, B & C and DrW antigens.

**Figure 1** Infiltration of the normal bronchus-associated lymphoid tissue by immunoblastic Non Hodgkin's Lymphoma. H and E × 250.

**Figure 2** Infiltration of the tracheal wall by Hodgkin's Disease beneath an intact surface epithelium. H and E × 165.

**Table III** Relative proportions of macrophages and lymphocytes from broncho alveolar lavage in lymphoma

| | | | | Percent | | |
|---|---|---|---|---|---|---|
| *Patient* | *Diagnosis* | *Smoking history* | *No. cells* | *macro-phages* | *T cells* | *B cells* |
| M.D. | HD $II_A$ NS | NS | $10^8$ | 27 | 53 | 4 |
| P.S. | HD $IV_A$ MC | S | $10^8$ | 92 | 6 | 1 |
| L.T. | HD $IV_B$ NS | S | $10^8$ | 92 | 2 | 1 |
| F.W. | HD $II_A$ NS | NS | $10^6$ | 75 | 20 | 2 |
| L.W. | HD $II_B$ NS | NS | $10^6$ | 70 | 21 | 5 |
| M.B. | NHL II Ib | S | $10^8$ | 80 | 10 | 2 |
| P.C. | NHL IV cc | S | $10^8$ | 90 | 5 | 1 |
| E.H. | NHL IV Cb | NS | $10^7$ | 70 | 25 | 3 |
| H.S. | NHL IV cc | S | $10^8$ | 90 | 5 | 1 |
| S.S. | NHL IV Ib | NS | $10^6$ | 65 | 29 | 6 |
| Controls (average of two) | | S | $10^8$ | 85 | 10 | 2 |
| Controls | | NS | $10^7$ | 65 | 20 | 5 |

For abbreviations see **Table II**.

### Discussion

Fibre optic bronchoscopy disclosed a higher incidence (8/25, 32%) of endobronchial disease at presentation in patients with malignant lymphoma than has previously been appreciated (Gribetz *et al.*, 1980; Phillips *et al.*, 1980).

The pattern of lymphomatous involvement varied with the histological type. All 3 patients with HD showed direct extension into the lumen from the surrounding nodal mass, whereas the 5 patients with NHL showed disseminated involvement of the "bronchus-associated lymphoid tissue" (Bienenstock, 1973) at each bronchial orifice. Patients with HD and a positive bronchoscopy, had lymphoma which was confined to the thorax, whilst in NHL all but one patient had widely disseminated disease outside the chest. Although there are reports of the diagnosis of both HD and NHL on sputum cytology (Giangreco *et al.*, 1980; Supron & Kross, 1964) these have all been in patients with advanced disease. By contrast in this group with newly diagnosed lymphoma the appearances at bronchoscopy, biopsy, and cytology all showed that the surface epithelium remained intact over the lymphomatous deposits.

Bronchoalveolar lavage has been used to investigate the pulmonary lymphoid tissue in both health and in a variety of diseases (Haslam *et al.*, 1980; Hunninghake *et al.*, 1979; Danielle *et al.*, 1977) but not previously in lymphoma. Alveolar macrophages and lymphocytes were present in normal proportions in lymphoma except in one patient with mediastinal nodular sclerosing HD. In this patient the Sternberg–Reed cells may have been shed from the associated infiltrating mass in the tracheal wall; however, the overlying respiratory epithelium was intact. Alternatively, they may have entered the alveoli from the blood stream as do the normal cellular constituents (Hocking & Golde, 1979; van Blusse and van Furth, 1979). This implies dissemination from the thoracic duct lymph which has been observed to contain Sternberg–Reed cells in association with mediastinal disease (Young, 1956; Watne *et al.*, 1960). In this case the peripheral blood was normal, and no other disease was found at staging laparotomy therefore treatment was given by mantle radiotherapy. However, relapse has occurred 16 months later with disseminated extranodal disease requiring chemotherapy.

In conclusion, bronchoscopy in newly diagnosed patients with malignant lymphoma with radiological evidence of ML demonstrated a previously unrecognised incidence of endobronchial disease which had arisen by direct infiltration in those with HD in comparison with lymphatic spread in those with NHL. If confirmed by further investigation these findings may provide an explanation for the worse prognosis of some patients with apparently localised Hodgkin's Disease (Mauch *et al.*, 1978; Lee *et al.*, 1980).

We are pleased to acknowledge the assistance of the Departments of Histopathology and Cytology at St Bartholomew's Hospital, Prof. A.E. Jones under whom some of these patients were admitted, and Dorothy Fletcher who typed the manuscript.

### References

BIENENSTOCK, J., JOHNSTON, N. & PEREY, D.Y.E. (1973). Bronchial lymphoid tissue I. Morphologic characteristics. *Lab. Invest.*, **28**, 686.

BLUSSE VAN OUD ALBAS A. & VAN FURTH, R. (1979). Origin, kinetics and characteristics of pulmonary macrophages in the normal steady state. *J. Exp. Med.*, **149**, 1504.

DANIELLE, R.P., DAUBAR, J.H., ALTOSE, M.D., ROWLANDS, D.T. & GORENBERG, D.J. (1977). Lymphocyte studies in asymptomatic cigarette smokers. *Am. Rev. Resp. Dis.*, **116**, 997.

DORREEN, M.S., HABESHAW, J.A., WRIGLEY, P.F.M. & LISTER, T.A. (1982). Distribution of T lymphocyte subsets in Hodgkin's Disease characterised by monoclonal antibodies. *Br. J. Cancer*, **45**, 491.

FILLY, R., BLANK, N. & CASTELLINO, R.A. (1976). Radiographic distribution of intrathoracic disease in previously untreated patients with Hodgkin's disease and non-Hodgkin's lymphoma. *Radiology*, **120**, 277.

GIANGRECO, A., ETTINGER, D.S., DRAGON, L.H., GUPTA, P.K. & LENHARD, R.E. (1980). Sputum cytologic diagnosis of Hodgkin's disease involving the lung. *Arch. Intern. Med.*, **140**, 910.

GOFFINET, D.R., WARNKE, R., DUNNICK, N.R. & 6 others. (1977). Clinical and surgical (laparotomy) evaluation of patients with Non Hodgkin's Lymphomas. *Cancer Treatment Rep.*, **61**, 981.

GRIBETZ, A.R., CHUANG, M.T. & TIERSTEIN, A.S. (1980). Fibreoptic bronchoscopy in patients with Hodgkin's and non-Hodgkin's lymphomas. *Cancer*, **46**, 1476.

HABESHAW, J.A. & YOUNG, G.A. (1975). Quantitation of subclasses of mononuclear cells in normal human blood by membrane receptor studies. *Br. J. Haematol.*, **29**, 43.

HASLAM, P.L., TURTON, C.W.G., HEARD, B. & 4 others. (1980). Bronchoalveolar lavage in pulmonary fibrosis: comparison of cells obtained with lung biopsy and clinical features. *Thorax*, **35**, 9.

HOCKING, W.G. & GOLDE, D.W. (1979). The pulmonar-alveolar macrophage. *N. Engl J. Med.*, **301**, 580.

HUNNINGHAKE, G.W., GADEK, J.E., KAWANAMI, O., FERRANS, N.J. & CRYSTAL, R.G. (1979). Inflammatory and immune processes in the human lung in health and disease; evaluation by bronchoalveolar lavage. *Am. J. Pathol.*, **97**, 149.

KAPLAN, H.S. (1980). *Hodgkin's Disease*. 2nd Ed., Harvard Univ. Press, Cambridge Mass. p. 333.

LEE, C.K.K., BLOOMFIELD, C.D., GOLDMAN, A.I. & LEVITT, S.H. (1980). Prognostic significance of mediastinal involvement in Hodgkin's Disease treated with curative radiotherapy. *Cancer*, **46**, 2403.

LISTER, T.A., CULLEN, M.J., BEARD, M.E.J. & 7 others. (1978). Comparison of combined and single agent chemotherapy in non-Hodgkin's lymphoma of favourable histology. *Br. Med. J.*, **i**, 533.

MAUCH, P., GOODMAN, R. & HELLMAN, S. (1978). The significance of mediastinal involvement in early stage Hodgkin's Disease. *Cancer*, **42**, 1039.

PECKHAM, M.J. (1973). Lung involvement. In: *Hodgkin's Disease* (Ed. Smithers) London: Churchill Livingstone, p. 118.

PHILLIPS, M.J., KNIGHT, R.K. & GREEN, M. (1980). Fibreoptic bronchoscopy and diagnosis of pulmonary lesions in lymphoma and leukaemia. *Thorax*, **35**, 19.

REYNOLDS, H.Y. & NEWBALL, H.H. (1974). Analysis of proteins and respiratory cells obtained from human lungs by bronchial lavage. *J. Lab. Clin. Med.*, **84**, 559.

ROBBINS, L.L. (1953). The Roentgenological appearance of parenchymal involvement of the lung by malignant lymphoma. *Cancer*, **6**, 80.

ROSENBERG, S.A., DIAMOND, H.D., JASLOWITZ, B. & CRAVEN, L.F. (1961). Lymphosarcoma: A review of 1269 cases. *Medicine*, **40**, 31.

STOLBERG, H.O., PATT, N.L., MACEWEN, K.F. & 2 others. (1964). Hodgkin's Disease of the lung. *Am. J. Roentgenol.*, **92**, 96.

SUGARBAKER, E.D. & CRAVEN, L.F. (1940). Lymphosarcoma: A study of 196 cases with biopsy. *J.A.M.A.*, **115**, 17.

SUPRUN, H. & KOSS, L.G. (1964). The cytological study of sputum and bronchial washings in Hodgkin's Disease with pulmonary involvement. *Cancer*, **17**, 674.

SUTCLIFFE, S.B., WRIGLEY, P.F.M., PETO, J. & 5 others. (1978). MVPP chemotherapy regimen for advanced Hodgkin's Disease. *Br. Med. J.*, **i**, 679.

VIETA, J.O. & CRAVEN, L.F. (1941). Intrathoracic manifestations of the lymphomatoid diseases. *Radiology*, **37**, 138.

WATNE, A.L., HATIBOGLU, I. & MOORE, G.E. (1960). A clinical and autopsy study of tumour cells in the thoracic duct lymph. *Surg. Gynaecol. Obstet.*, **110**, 339.

YOUNG, J.M. (1956). The thoracic duct in malignant disease. *Am. J. Pathol.*, **32**, 253.